# Quantitation and pharmacokinetic modeling of therapeutic antibody quality attributes in human studies

**DOI:** 10.1080/19420862.2016.1186322

**Published:** 2016-05-24

**Authors:** Yinyin Li, Michael Monine, Yu Huang, Patrick Swann, Ivan Nestorov, Yelena Lyubarskaya

**Affiliations:** Biogen, Cambridge, MA, USA

**Keywords:** Affinity purification, clinical studies, human studies, in vivo, mass spectrometry, PTM, quality attributes, therapeutic antibody

## Abstract

A thorough understanding of drug metabolism and disposition can aid in the assessment of efficacy and safety. However, analytical methods used in pharmacokinetics (PK) studies of protein therapeutics are usually based on ELISA, and therefore can provide a limited perspective on the quality of the drug in concentration measurements. Individual post-translational modifications (PTMs) of protein therapeutics are rarely considered for PK analysis, partly because it is technically difficult to recover and quantify individual protein variants from biological fluids. Meanwhile, PTMs may be directly linked to variations in drug efficacy and safety, and therefore understanding of clearance and metabolism of biopharmaceutical protein variants during clinical studies is an important consideration. To address such challenges, we developed an affinity-purification procedure followed by peptide mapping with mass spectrometric detection, which can profile multiple quality attributes of therapeutic antibodies recovered from patient sera. The obtained data enable quantitative modeling, which allows for simulation of the PK of different individual PTMs or attribute levels *in vivo* and thus facilitate the assessment of quality attributes impact *in vivo*. Such information can contribute to the product quality attribute risk assessment during manufacturing process development and inform appropriate process control strategy.

## Introduction

A thorough understanding of drug metabolism and disposition can aid in the assessment of efficacy and safety.^1-4^ For small molecule pharmaceuticals, the identities and pharmacokinetics (PK) of individual drug metabolites, in addition to the drug itself, are routinely monitored during clinical trials.^1-4^ In contrast, the PK of biopharmaceutical proteins is by in large limited to the total drug concentration measurement.^5,6^ Monitoring of individual metabolites/modifications of protein therapeutics in vivo is rarely performed, and the effect on patient exposure is not considered. This limitation is partly due to the complex micro-heterogeneity of biopharmaceuticals and their similarity to endogenous proteins, which makes it difficult to recover and quantify individual protein variants.^5,6^

Despite these technical difficulties, the value of investigating the behavior of different quality attributes in vivo is gaining increasing attention.^7-20^ Through such investigations, the PK effect of different modifications, such as deamidation, high mannose, oxidation, disulfides, can be elucidated.^7-20^ Many of these modifications are also formed during manufacturing, and thus must meet certain criteria, ranges or specifications. By quantifying the PK effect of an individual quality attribute, it becomes possible to establish meaningful manufacturing ranges that are supported by clinical evidence.^21^ Moreover, monitoring individual variants of biopharmaceuticals in vivo enables quantitative modeling, which allows simulation of the effect of different levels of quality attributes on patient exposure.^21^ Such information can facilitate risk assessment during manufacturing process development, and inform appropriate process control strategy. Several studies have been reported to date, mostly focusing on certain quality attributes of monoclonal antibodies during PK studies in animal or human subjects. ^7-20^ More recently, we reported a study in which simultaneous monitoring of multiple attributes of a therapeutic IgG4 monoclonal antibody in cynomolgus monkeys was performed.^22^ There studies contribute to a quantitative assessment of impacts of individual therapeutic protein variants in vivo.

Building upon previous experience,^22^ we extended our multiple-attribute profiling to human PK studies. To reduce the endogenous protein background, we utilized an affinity purification procedure using a capturing antibody that can specifically recognize the unique complementarity-determining region (CDR) of a therapeutic human IgG1 MAB2, along with magnetic beads with low non-specific binding properties.^23-25^ After the affinity purification, liquid-chromatography-mass spectrometry (LC-MS) peptide mapping analysis was utilized to simultaneously quantify multiple quality attributes from MAB2 in 3 human subjects. Based on the experimental data, quantitative PK models were built for the quality attributes of interest. To demonstrate the utility of this approach, representative models are shown here for 2 attributes, an Fc deamidation site and Man5 glycoform. The Fc deamidation site demonstrated relatively rapid formation in vivo, while Man5 glycoform showed rapid clearance. To further explore the models, we simulated the exposure impact by assuming different original levels of these attributes in MAB2.

## Results

**Figure 1.**
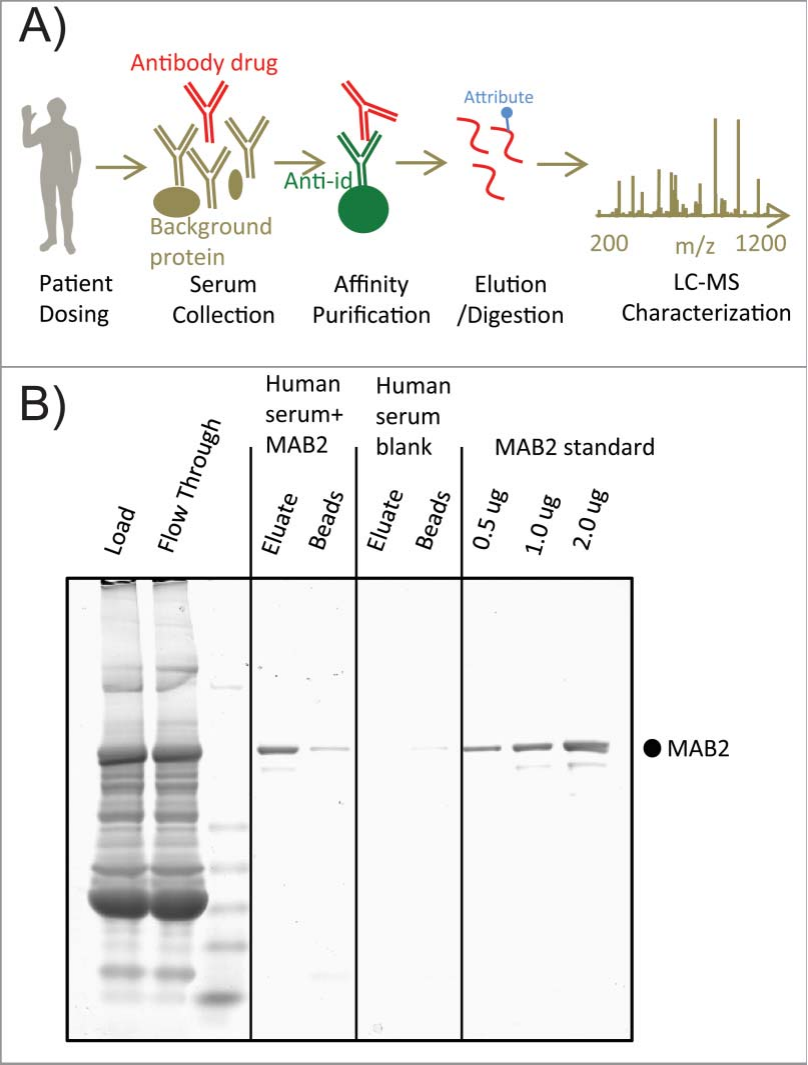
Affinity purification of MAB2 from human serum. (A) Scheme of affinity-purification coupled with mass spectrometric analysis for profiling quality attributes of antibody drug from patient samples. (B) SDS-PAGE analysis of affinity purified spike-in samples and blank control. One ug MAB2 was spiked into human serum and subsequently recovered by affinity purification. The gel was stained by Coomassie blue.

Analysis and quantitation of individual antibody attributes from patient serum require quantitative and unbiased recovery of antibody related variants. Because it is difficult to analyze protein therapeutics directly from biological fluids, specificity and selectivity of a purification procedure are essential for obtaining good-quality data. Affinity purification is a tool providing excellent selectivity and specificity for extracting therapeutic antibody molecules from serum, especially in the presence of similar endogenous IgGs (Fig. 1A). The key element of efficient affinity purification is a capturing reagent.^25^ A high affinity antigen is frequently used as a capturing reagent for therapeutic antibodies. ^8^ However, in this case, the MAB2 antigen is complex and not suitable for affinity purification purposes. A mouse anti-idiotypic (anti-id) antibody was generated against human IgG1 MAB2. The anti-id binds to the signature CDR regions of MAB2 with high affinity and excellent specificity, which allows MAB2 to be distinguished from endogenous IgGs and other background proteins (Fig. 1B).

**Figure 2.**
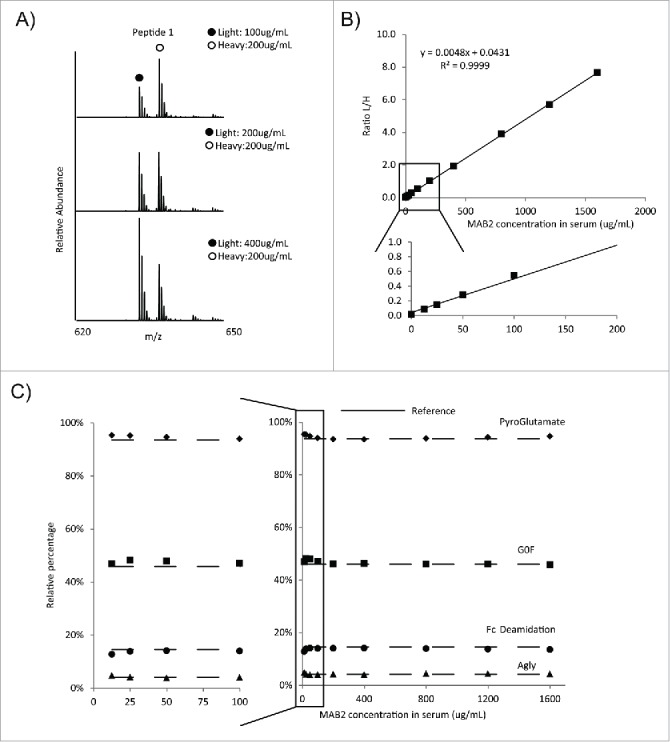
MS based quantitation of MAB2 purified from spike-in standards. Increasing amounts of MAB2, along with a constant amount of heavy MAB2 calibrant, were spiked into human serum, and recovered by affinity purification for Lys-C peptide mapping with LC-MS. (A) Mass spectra of a representative peptide. (B) One representative standard curve. The standard curve was generated by plotting peak area ratios of light over heavy peptides against spike-in MAB2 concentration. (C) Relative levels of quality attributes of MAB2 purified from spike-in standards. The relative percentages of quality attributes of interests were plotted against spike-in MAB2 concentration. The dashed line represents the percentage of attributes in MAB2 reference sample and not subjected to affinity purification.

Absolute quantitation of peptides obtained after digestion of an antibody affinity purified from serum may be quite challenging. To account for any potential bias of the method, including antibody recovery during affinity purification, enzymatic digestion of the purified protein at different concentrations, as well as LC-MS analysis of the peptides present in different samples at different levels, it is important to select an appropriate standard. While an external calibration can be conducted and used for further quantitation, it is best to utilize an internal calibrant, which is added to the sample, that undergoes the same handling as the molecule of interest, and behaves as the molecule of interest during both affinity purification and LC-MS analysis. A heavy isotope-labeled MAB2 was chosen as an ideal internal calibrant for absolute quantitation. In the heavy-labeled MAB2 internal calibrant, all lysine and arginine residues were substituted by their ^13^C and ^15^N containing counterparts (purity = 99%). This internal calibrant provided a way to normalize method-related variability by calculating the ratio of a “light” peptide (from circulating MAB2) and a “heavy” peptide (from the spiked-in internal calibrant). Previously, we used parallel reaction monitoring (PRM) to quantify peptides using fragment ions from a single parent as a way to further reduce the interference after the affinity purification.^22^ In this study, due to the high specificity of affinity purification provided by the anti-id (Fig. 1B), MS quantitation based on extracted ion chromatograms (XIC) from full scan MS analysis was sufficient to avoid interferences and provide reliable quantitation over the concentration range of the PK samples (Fig. 2). In addition to high resolution full scan MS, the LC-MS method included sequential tandem MS fragmentation of the 3 most abundant precursor peaks (“Top3” method). One advantage of this method compared to PRM is that new or unexpected modifications can be detected and quantified, while the PRM method presumes creation of a “target list," where the expected peptide ions to be fragmented are listed. The affinity purification coupled LC-MS method demonstrated excellent linearity (R^2^ = 0.9999) (Fig. 2B), even at low MAB2 concentration (Fig. 2B inlet). In addition, it was shown that relative quantitation of individual quality attributes was consistent across a wide range of concentrations (Fig. 2C). The level of attributes from affinity-purified MAB2 was determined to be in agreement with that of a reference MAB2 not subjected to affinity purification (Fig. 2C). This result demonstrated that the affinity purification/ LC-MS approach had no significant bias to a particular PTM, and thus was appropriate for quantitative analysis of attributes of interest.

**Figure 3.**
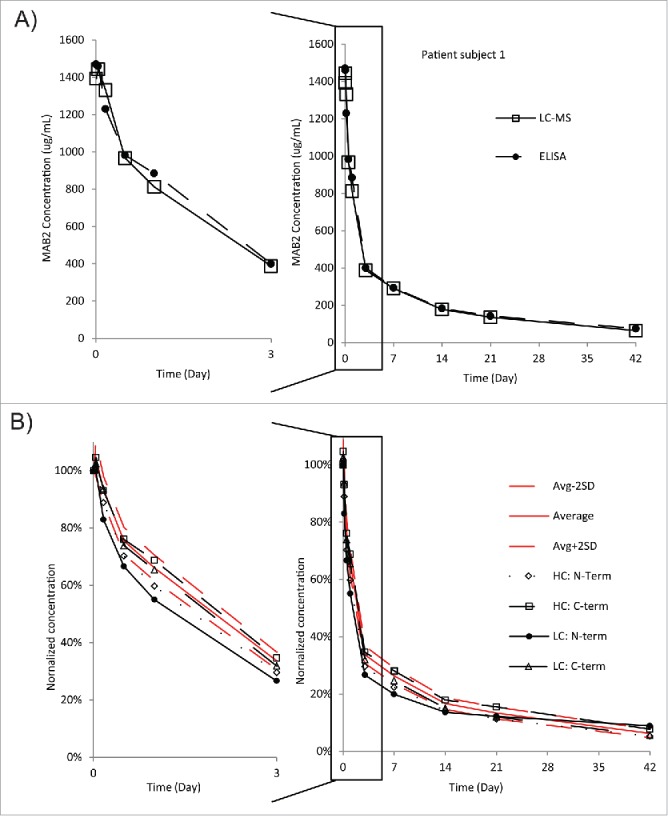
PK measurements of MAB2 from patient serum samples. (A) Comparison of ELISA and affinity-purification LC-MS data obtained for patient serum samples. The MS data points represent the average concentrations of 20 selected Lys-C digested MAB2 peptides. The selection criteria were described in the Results section. (B) In vivo dynamics of terminal peptides. PK profiles of terminal peptides were overlaid onto the average profiles of 20 peptides to identify outliers. The selection criteria of these 20 peptides were described in the Results section.

We chose to designate an average concentration of 20 peptides as the representative of the total protein concentration. These 20 peptides were selected based on the following criteria: 1) high intensity; 2) absence of carryover and modifications. The quantitative results obtained from LC-MS were consistent with the data generated in ELISA-based analysis (Fig. 3A). In order to determine the concentration and behavior of different MAB2 variants over the course of the PK study at the peptide level, a normalized concentration (PK) curve can be plotted for any peptide of interest and compared with the total MAB2 concentration (PK) curve. For example, N- and C-terminal peptide concentrations were plotted as a time course and compared to the total MAB2 concentration, based on the 20-peptide average (Fig. 3B). This approach allows us to assess whether an N- or a C-terminal degradation occurs in vivo. Indeed, it was determined that the N-terminal light chain peptide concentration was decreasing faster than the total MAB2 concentration over the course of the PK study (Fig. 3B). This finding suggests that the N terminus of the light chain is subjected to preferential degradation in vivo.

**Figure 4.**
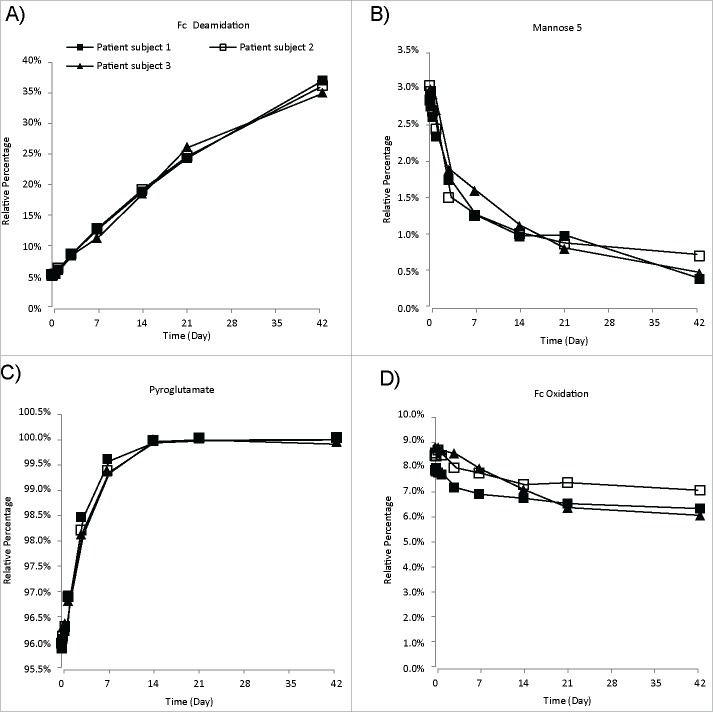
In vivo dynamics of quality attributes from 3 different patient subjects. The relative percentage of targeted quality attributes were plotted against sample collection time. The data were from 3 different patient subjects with the same MAB2 dosage. (A) An Fc deamidation site. (B) Mannose 5-containing Fc glycoform. (C) Heavy chain N-terminus Pyroglutamate. (D) An Fc methionine oxidation site.

To illustrate in vivo dynamics of MAB2 variants containing different modifications or attributes, the time course for relative levels of multiple quality attributes was plotted for the first 6 weeks (42 days) of the SAD PK study. While the samples in this PK study were collected over 24 weeks, the 6 week time period was selected, on the one hand, considering the sensitivity and linearity of our methodology. On the other hand, this time frame provides adequate information for the purposes of our investigation, as 6 weeks is approaching 3 half-lives of MAB2 (t_1/2_ = 18 Days), which is sufficient for trending and modeling the behavior of quality attributes in vivo. The time course plots for 4 representative attributes are shown in Fig. 4. The most susceptible deamidation site, a conserved site in the Fc domain that is commonly found in human and humanized monoclonal antibodies (mAbs), demonstrated a rapid increase in deamidation from 5% to 35% over 6 weeks (Fig. 4A). Rapid increase in deamidation levels in vivo has been previously reported, and can be caused by physiological pH and temperature.^19^ In contrast, the Man5-containing glycoform of MAB2 decreased from 3% to 0.5% within 6 weeks (Fig. 4B), while other glycoforms containing neutral complex glycans remained relatively unchanged (data not shown). This finding is in agreement with current understanding of glycoprotein clearance in serum.^10^ As proposed, the Man5 level demonstrates faster clearance due to a high-mannose receptor-mediated clearance pathway.^26^ A complete conversion of N-terminal glutamine residue into N-terminal pyroglutamate, from 96% to 100% (Fig. 4C), was observed in vivo. It was found that the oxidation level of an Fc methionine decreased slightly (Fig. 4D), from ∼8.5% to ∼7%, which may be indicative of a slight preferential clearance of Fc-oxidized mAbs.^18^ It is also worth noting that most of the MAB2 quality attributes remained unchanged (data not shown), indicating that therapeutic IgG1s are quite stable in human circulation. It was also found that the behavior of MAB2 variants in vivo is similar across 3 patients (Fig. 4).

**Figure 5.**
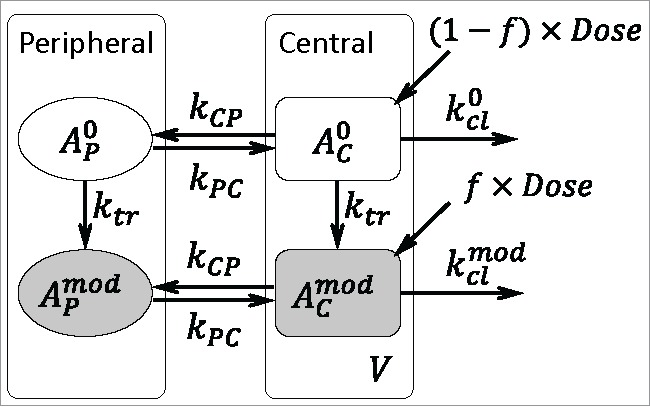
Scheme of the mechanistic PK model for individual quality attributes. The same modeling framework is applicable to both the deamidation and Man5 models.

To evaluate the effect of MAB2 variants with different modifications or attributes on patient exposure, we performed a mechanistic PK modeling analysis. Understanding how PK properties of the therapeutic antibody can potentially vary depending on the level and the behavior of an attribute or a modification in vivo is critical for predicting the patient exposure to an attribute, and its potential impact on safety or efficacy. Here, the 2 representative attributes selected for PK modeling were a conserved Fc deamidation site and Man5 containing glycoform in the Fc region (Figs. 4A and B). First, the data for both attributes was fitted with the corresponding PK model (Fig. 5). The best fit parameters for the deamidation and Man5 models are summarized in Table 1. The two models were then used to predict time-dependent concentrations in vivo of MAB2 with different initial levels of deamidation and Man5.

**Table 1.** Parameters of PK models for deamidation and Man5.

Parameter	Description	Deamidation model	Man5 model
$k_{CP}$, d^-1^	Transition from central to peripheral compartment	0.17 (35.9 %)*	0.18 (57.4 %)
$k_{PC}$, d^-1^	Transition from peripheral to central compartment	0.093 (1.4 %)	0.096 (7.3 %)
$k_{tr}$, d^-1^	Transition from original to modified form	0.01 (3.1 %)	0
$k_{cl}^{0}$, d^-1^	Clearance of the original form	0.13 (10.8 %)	0.127 (4.6 %)
$k_{cl}^{mod}$, d^-1^	Clearance of the modified form	0.13 (10.8 %)	0.295 (8.1 %)
V , L	Distribution volume of central compartment	3.34 (26.2 %)	3.32 (28.6 %)

*The numbers in parenthesis denote coefficients of variation (CV) of parameter estimates calculated as CV = Standard deviation / Average * 100%.

**Figure 6.**
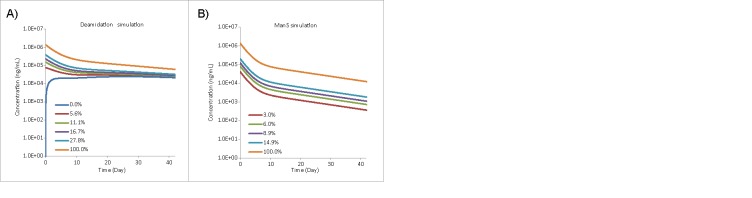
Simulated PK curves of 2 quality attributes. (A) Simulated PK curves of Fc deamidation at different initial levels (pre-administration). (B) Simulated PK curves of Man5 glycoform at different initial levels (pre-administration).

As only one batch of MAB2 was used in this clinical study, experimental PK data available for our analysis have the identical initial levels of deamidation and Man5. As a result, direct validation of model predictions for other initial levels was not possible. We hypothesize that differences in half-life of each modification can be attributed to the clearance and conversion rates. Earlier studies of IgG deamidation at the conserved Fc site suggest that the deamidation process takes place with the same rate in vivo and under physiologically-relevant conditions in vitro.^16^ In the absence of the antibody clearance factor in vitro, it is safe to conclude that the conversion rate is the main factor driving deamidation both in vivo and in vitro, and there is no reason to invoke differential clearance rates for the deamidated and non-deamidated forms in vivo. Therefore, the deamidation model built on this hypothesis predicts accumulation of the deamidated form over 42 d even if the initial level of deamidation in the MAB2 dose administered is 0% (Fig. 6A). As shown in Table 2, the area under the concentration-time curve (AUC) for the deamidated species is increasing at half the rate as initial deamidated level increase. A 2-fold increase in initial deamidation level (from 5.6% to 11.2%) leads to the increase of deamidation-specific AUC by only a third (from 15.4% to 20.4%). Thus, the deamidation level in vivo is driven mostly by conversion rather than by the initial level.

**Table 2.** Simulation results of 2 representative quality attributes.

Attribute	Initial level (%)	Attribute AUC* (hXmg/mL)	Total AUC (hXmg/mL)	Relative AUC** (%)
Deamidation	0.0	21.9	209.7	10.5
	5.6	32.4	209.7	15.4
	11.2	42.8	209.7	20.4
	27.8	74.2	209.7	35.4
Man5	0.0	0.0	212.8	0.0
	3.0	3.2	209.6	1.5
	6.0	6.4	206.4	3.1
	14.9	15.9	196.9	8.1

*AUC: area under curve integrated from Day 0 to Day 42.

**Relative AUC = (Attribute AUC / Total AUC)×100%.

In contrast to deamidation, the Man5 conversion (formation) is intracellular and is negligible during circulation,^27^ and the Man5 glycoform demonstrates faster clearance possibly due to a high-mannose receptor-mediated clearance pathway.^28^ To reflect this hypothesis in the Man5 model, the Man5 conversion rate is set to zero and the Man5 PK data is fitted by adjusting the clearance rate constants for the non-modified and modified forms ($k_{cl}^{0}$ and $k_{cl}^{mod}$, respectively). The estimated value of the modified clearance rate constant for the Man5 variant ($k_{cl}^{mod}$) is 2.3 times higher than those for other forms of MAB2 (Table 1), which is consistent with previous findings.^10^ Therefore, we conclude that the in vivo concentration of the Man5 modification is controlled by both the initial level and the clearance rate (Fig. 6B and Table 2). Evidently, if the level of Man5 glycoform is increased upon manufacturing, this could have a significant effect on the overall MAB2 PK profile.

## Discussion

In biopharmaceutical product development and manufacturing, substantial effort is focused on designing and developing processes that reproducibly deliver product quality attributes within predefined targets. Especially important consideration is given to critical quality attributes (CQAs), which can affect safety, efficacy or clearance of therapeutic biologics.^28^ The identification of CQAs for biopharmaceutical products can be challenging, in particular due to their structural complexity and heterogeneity. As therapeutic antibodies and recombinant proteins possess a large number of variants and modifications or quality attributes, it is often not feasible to fully evaluate the impact of each one. It is important to minimize such uncertainties in the CQA identification process in order to develop a robust and scientifically justified control strategy, which enables consistent manufacture of safe and efficacious biologics.^29^ Understanding product attribute criticality requires integration of data and knowledge from multiple sources, including, structure-activity relationship studies, preclinical and clinical experience. Clinical experience is clearly the major source of in vivo information regarding safety, efficacy and clearance of a biopharmaceutical product. However, relevant experience in human subjects is often limited to a few clinical batches of material, which leads to narrow limits and specifications for product quality attributes. In addition, understanding the behavior and composition of individual variants or attributes of antibody or other protein-based therapeutics in vivo is not widely studied. In general, extensive studies of a pharmaceutical metabolism or conversion and clearance or elimination in vivo, are well established and routinely performed for small molecule therapeutics. Thus, small molecule drugs have less residual uncertainty in CQA identification, in part because their behavior in patients is generally well understood. This also highlights the need for improved assessment and understanding of metabolism and clearance of biopharmaceutical product variants and individual quality attributes in patients.

In this study, we devised an approach for investigation of the quality attributes of a therapeutic antibody from patient samples. The major technical hurdle was availability of affinity capturing reagents that exhibit high specificity and affinity. A common affinity-purification approach is using a recombinantly produced antigen as capturing reagent.^8,11,12^ However, this approach is not applicable to MAB2 due to the complexity of its antigen. It has also been reported that multiple reaction monitoring (MRM) methods can quantify therapeutic antibody from human serum samples without any enrichment steps.^30^ Although such an approach carries lower risks of bias and is easier to develop, MRM methods can only quantify peptides with unique sequences, such as CDR peptides, and are not suitable for quality attribute profiling of constant regions. In this study, we used an anti-idiotypic antibody that was developed in mouse as capturing reagent. It recognized the CDR regions of MAB2 and demonstrated high specificity toward MAB2 in human serum.

The results shown in this study are qualitatively similar to our previous cynomolgus monkey study of an IgG4 for the attributes common between the 2 molcules.^22^ This suggests that non-human primate data may be predictive for clinical studies in human subjects for common quality attributes of therapeutic IgGs. These findings also propose that different IgG subclasses (IgG4 and IgG1) behave similarly in terms of some quality attributes dynamics in vivo. The ability to generalize these findings to all other IgG antibodies is limited. Clearance and distribution patterns might widely differ depending on the variety of factors, including site-specific and unique modifications of IgGs, location and distribution of therapeutic targets, mechanism of action, dosage and administration regiment.

Finally, the trends from 3 different subjects were similar for the same attribute. While collecting data from a larger patient population, as well as potentially exploring different dose regiment and route of administration, may be beneficial, our current findings suggest that the in vivo dynamics of these 4 mAb quality attributes are not apparently patient-specific.

Based on the mechanistic PK models built upon the experimental data, it becomes possible to simulate the in vivo effects of attribute level fluctuation caused by process change or lot-to-lot variability. For example, the modeling showed that the effect of in vivo deamidation is relatively insensitive to the initial attribute level. It provides a scientific justification for widening the acceptable ranges for this attribute during manufacture and assuring comparability. Generally, widening acceptable ranges for a variety of attributes, based on better understanding of attribute criticality, will facilitate process changes and alleviate comparability concerns.

In addition to affecting risk assessment, process control and comparability consideration aspects of biopharmaceutical development and manufacture, we expect that similar studies will play an important role in drug design, and bio-similar development. With automation, increased throughput and streamlining data processing and PK modeling, investigation of product quality attributes in vivo is expected to be more widely found in clinical programs, supplementing results derived using traditional ELISA methods.

## Methods

### Reagents

Mass spectrometry grade lysyl endopeptidase (125-05061) was purchased from Wako Chemicals (Richmond, VA); dithiothreitol (DTT, 45779) was purchased from Sigma-Aldrich (St. Louis, MO). Phosphate-buffered saline (PBS, 10010023), LC/MS grade water (W5), HPLC grade acetonitrile (ACN, A998), trifluoroacetic acid (TFA, 28904) were obtained from Fisher Scientific (Fairlawn, NJ). Sterile human serum (D119-00-0100) was purchased from Rockland Immunochemicals (Gilbertsville, PA). Epoxy dynabeads (14301) were purchased from Life Technologies (Chicago, IL). MAB2 was produced by Biogen Manufacturing. Internal MAB2 calibrant (heavy-labeled MAB2) was produced by Biogen Cell Culture Development.

### Clinical sample information

The clinical samples are from a single ascending dosage (SAD) PK study in patients. Three patient subjects were given a single high dose of MAB2 intravenously. The blood samples were collected at designated time points and processed into serum for storage at -70 °C. The clinical study protocols were reviewed and approved by the local institutional review boards. The studies were conducted in accordance with regulatory guidelines. Written informed consents were obtained from all participants. Samples from 11 time points were subjected to quality attribute profiling: pre-bleed, 10min, 1h, 4h, 12h, Day2, Day4, Week1, Week2, Week3, and Week6. Standard solutions were made by spiking MAB2 into blank human serum, and serially diluted with serum.

### Affinity purification

Mouse anti-idiotypic antibody against MAB2 was conjugated to epoxy magnetic dynabeads as previously reported.^24^ The conjugated beads were incubated with patient serum (12.5 μL) diluted in PBS buffer (400 μL), along with MAB2 internal calibrant (2.5 μg), containing heavy-labeled lysine and arginine residues. After 1h incubation on a rotator at room temperature, the beads were washed 3 times with PBS (1 mL each). After the wash, bound MAB2 was eluted and digested with LysC (1 μg) at 25°C for 20 h. The digested samples were stored at -70 °C prior to analysis.

### LC-MS

The digested samples were thawed at room temperature and supplemented with DTT to 10 mM. Peptides were then separated on a reverse phase C18 column (2.1 × 100 mm, Waters Acquity BEH H3 TSS) on an Agilent 1200 HPLC system at a flow rate of 0.4 mL/min, with a 50 min linear gradient from 0% to 45% acetonitrile. The column temperature was 55 °C. 0.1% TFA was added as ion pairing reagent to both mobile phase A and B to improve chromatographic behaviors of peptides, at an affordable cost of MS sensitivity. A Thermo HESI source was used for peptide ionization on-line with HPLC. The MS detection was carried out by a Thermo Orbitrap Discovery instrument with a standard “Top 3” method: a high resolution full scan (R = 30K) in the orbitrap followed by 3 tandem MS scans of 3 most abundant precursor peaks in the ion trap.

### MS data processing

MS raw data were processed by Thermo PinPoint software (Version 1.4). A processing template was created to identify lysine C-digested peptides from the MAB2 sequence. Both modified (PTM-containing) and unmodified peptides were included in the template, according to their precursor mass to charge ratios (m/z) and retention time. The identity of peptides can be further checked by visually examining co-elution of multiple charge species and isotopic distribution. In some cases, tandem MS information was utilized to confirm the identity, in either Thermo PepFinder or Proteome Discover software. After Pinpoint processing, the extracted ion chromatograms (XIC) of multiply charge ions were created, and areas under the resulting peaks were integrated and added together. Subsequently, the peak areas were exported to a table in CSV format. The table was then transferred into a macro-enabled Excel template built in-house. The template automatically groups the result for peak areas of the same peptide with various modifications. Relative percentage of each quality attribute was calculated by dividing the peak area of a modified peptide over the sum of the peak areas of all the forms (modified and non-modified) of this peptide. For calculating the absolute concentration, peak area ratios of “light” peptides over their “heavy” counterparts were obtained in a similar manner from the PinPoint template. These quantitative results, in combination with collection time, were used for PK modeling.

### Pharmacokinetic modeling

A PK model was built based on the experimentally obtained quantitative data for metabolism or clearance of individual PTMs/quality attributes. The model allows for assessment of a patient exposure to individual quality attributes at various initial levels of an attribute/modification. Two representative attributes were selected: a conserved Fc deamidation site and Man5 containing glycoform. Peptide mapping analysis allowed for quantitation of individual attributes at the peptide level, but any potential attribute interactions and their combinatory effect on MAB2 PK could not be accounted for. Thus, 2 simplified attribute-specific models have been built to describe deamidation and Man5 modification individually, while any potential attribute interactions were neglected. A generic model structure that applies to both attributes is shown in Fig. 5. For each attribute, 2 MAB2 variants are considered: original (non-modified) and modified, each of which is described by a standard 2-compartment model. This type of models is known to best represent biologics PK in vivo, as it accounts for a physiologically relevant distribution phase (between central/plasma and peripheral/tissue compartments), and elimination phase (from central/plasma compartment).^31^ Each attribute can potentially undergo conversion from the original form to the modified form in vivo. Mathematically, a generic model can be expressed as a system of coupled ordinary differential equations,

(1) $$\frac{dA_{C}^{0}}{dt}=-\left(k_{CP}+k_{tr}+k_{cl}^{0}\right)A_{C}^{0}+k_{PC}A_{P}^{0},$$

(2) $$\frac{dA_{P}^{0}}{dt}=-\left(k_{PC}+k_{tr}\right)A_{P}^{0}+k_{CP}A_{C}^{0},$$

(3) $$\frac{dA_{C}^{mod}}{dt}=-\left(k_{CP}+k_{cl}^{mod}\right)A_{C}^{mod}+k_{tr}A_{C}^{0}+k_{PC}A_{P}^{mod},$$

(4) $$\frac{dA_{P}^{mod}}{dt}=-k_{PC}A_{P}^{mod}+k_{tr}A_{P}^{0}+k_{CP}A_{C}^{mod},$$

where variables $A_{C}^{0}$, $A_{P}^{0}$, $A_{C}^{mod}$ and $A_{P}^{mod}$ denote the amounts of the original attribute form in the central and peripheral compartments and the modified attribute form in the central and peripheral compartments, respectively. The model parameters are defined in Table 1. All terms in Eqs. 1-4 imply first order transitions/reactions in vivo. To relate the simulated amounts to the observed concentrations in plasma, a distribution volume, V, was estimated and assumed to be the same for both the original and modified attribute forms. Since the initial amount of MAB2 administered via IV can contain both the original and modified attribute forms, the model has 2 inputs to the central compartment, $A_{C}^{0}\left(t=0\right)=\left(1-f\right)\times Dose$ and $A_{C}^{\text{mod}}\left(t=0\right)=f\times Dose$. Here, *Dose* is the total amount administered and *f* is the initial fraction of the modified form included in the IV dose. The quantity 100×f is referred to as the “initial level” of an attribute (modification). The generic model was further modified to describe the PK of drug variants with specific attribute. Several assumptions were built into the attribute-specific models: 1) The Fc site deamidation has no impact on clearance^16^, i.e., $k_{cl}^{mod}=k_{cl}^{0}$; 2) Man5 does not form during circulation^27^, i.e., $k_{tr}=0$; 3) Man5 containing variant has faster clearance^10^, i.e., $k_{cl}^{mod}>k_{cl}^{0}$; 4) the nature of an attribute has no effect on the distribution rates between central and peripheral compartments, i.e., the rate constant for transition from central to peripheral compartments, $k_{CP}$, is the same for both the original and modified attribute forms, and similarly the rate constant for transition from peripheral to central compartments, $k_{PC}$, is the same for both forms. Estimation of individual parameters was done in ADAPT.^32^ The best fit parameter estimates are given in Table 1.
